# Cutaneous vs. Mucosal Tropism: The Papillomavirus Paradigm Comes to an “*and*”

**DOI:** 10.3389/fmicb.2020.588663

**Published:** 2020-10-14

**Authors:** Gennaro Altamura, Massimo Tommasino, Giuseppe Borzacchiello

**Affiliations:** ^1^Department of Veterinary Medicine and Animal Productions, University of Naples Federico II, Naples, Italy; ^2^Infections and Cancer Biology Group, International Agency for Research on Cancer (IARC), Lyon, France

**Keywords:** papillomavirus, tropism, epithelia, mucosa, skin, HPV, FcaPV, EcPV

## Introduction

Papillomaviruses (PVs) are small, double stranded DNA viruses with a preferential tropism for epithelial tissues (Van Doorslaer et al., 2018). Approximately 300 PV types have been isolated so far and placed in the taxonomy, however several studies indicate that many more PV types exist (Araldi et al., 2017; Brancaccio et al., 2018; Galati et al., 2020). The canonical criteria for the placement of new isolates in the phylogenetic tree, which are based on the percentage of similarity with other known PVs within the L1 gene sequence, establish that a PV is classified as a new viral type when the difference is >10% (de Villiers, 2013). All PV types belong to the family of *Papillomaviridae* which, according to the most recent classification, is split in two subfamilies sharing <45% of sequence identity in L1, namely *Firstpapillomavirinae* and *Secondpapillomavirinae* (Van Doorslaer et al., 2018). Subfamilies are divided in subgroups called genera (α, β, γ, and so on), which are further subdivided into species (numbered as 1, 2, 3, and so on) (de Villiers, 2013). Each of the genera can include animal and human PVs (HPVs), which are numbered consecutively in order of discovery. *Secondpapillomavirinae* currently includes a single genus (*Alefpapillomavirus)* and a unique viral species, isolated from a skin lesion of a fish (gilt-head sea bream, *Sparus aurata*) (Van Doorslaer et al., 2018).

Beyond the conventional criteria for classification according to L1 sequence, non-canonical ways based on biological properties have become of common use. For instance, mucosal α-HPVs comprise high-risk (HR) or low-risk types, according to their role in causing malignant or benign lesions, respectively (Zur Hausen, 2002).

In addition, PVs can be classified as cutaneous or mucosal, based on their ability to infect cutaneous or mucosal epithelia (de Villiers, 2013). Over decades of research, this tropism-based nomenclature has become almost a paradigm, which has led to PVs affinity for skin or mucosal epithelia being considered as mutually exclusive (de Villiers, 2013).

However, a series of recent studies indicate that specific subgroups of animal and human PVs could retain a dual tropism.

## Breaking The Paradigm: Recent Evidence About Animal and Human PVs

Studies of feline PVs indicate that the same virus can be associated with development of cancers of the skin and mucosal epithelia. *Felis catus* PV type 2 (FcaPV-2), originally isolated from and then widely associated with pre-neoplastic and neoplastic skin lesions, is detectable in a subset of feline oral squamous cell carcinoma (FOSCC) samples and FOSCC-derived cell lines, where it is transcriptionally active (Lange et al., 2009; Altamura et al., 2018a, 2020; Munday et al., 2018). Accordingly, molecular studies clearly show that FcaPV-2 E6 and E7 oncogenes display biological properties similar to those of mucosal HR HPV-16 in degrading p53 and pRb tumor suppressor proteins (Altamura et al., 2016, 2018a). Possible transmission routes between the two anatomical sites have also been hypothesized, such as via skin licking or through the bloodstream carrying viral particles to the epithelium of oral cavity (Altamura et al., 2018b, 2020). Of note, other FcaPVs are suspected to infect skin and oral epithelia, such as FcaPV-1, -3, and -4 (Munday and French, 2015; Munday et al., 2015, 2018; Mazzei et al., 2018; Vascellari et al., 2019; Chu et al., 2020), however additional experimental data are warranted for any confirmation.

New evidence also comes from *Equus caballus* PV type 2 (EcPV-2). It is recognized as the causative agent of neoplastic lesions arising on the skin of external genitalia of the horses; indeed, EcPV-2 DNA and mRNA are consistently found in equine penile SCC and relative precursors (Sykora and Brandt, 2017). More precisely, EcPV-2 induced diseases develop preferentially at mucocutaneous junctions, suggesting a possible affinity for cells of both the skin and mucosal epithelia of genital region (Sykora and Brandt, 2017; Ramsauer et al., 2019). Interestingly, the presence of EcPV-2 has also been reported in a subset of equine head and neck cancers (HNCs), and recent work shows that EcPV-2 is detectable in neoplastic cells of equine gastric SCC (Knight et al., 2013; Sykora et al., 2017; Alloway et al., 2020).

It is also worth mentioning the growing body of evidence for expanded tissue tropism exhibited by the recently discovered *Mus musculus* PV (MmuPV-1). Similarly to HR-HPVs, MmuPV-1 is sexually transmitted, however it can spread from ano-genital mucosa to different cutaneous sites and *vice versa* and cause development of neoplastic lesions in all of these anatomical regions (Cladel et al., 2017; Spurgeon and Lambert, 2019). Interestingly, MmuPV-1 E6 and E7 retain several molecular activities associated with cell transformation by mucosal HR-HPVs, despite the limited similarity in the nucleotide sequence of their respective oncogenes (Spurgeon and Lambert, 2020).

Studies of HPVs confirm that some viral types have a dual tropism. HPVs of genus β are classified as cutaneous by assumption, given that most, if not all, of them have been isolated from the skin (Quint et al., 2015). β-1 and β-2 HPV types are suspected to be major players in the development of cutaneous SCC in cooperation with UV exposure, particularly in patients with immunosuppressive disorders and transplants recipients (Bandolin et al., 2020). However, a series of reports show that DNA from β-HPVs can be consistently found in other anatomical sites in addition to skin, such as external genital skin, nasal cavity, anal and oral mucosa, where their presence is even associated with increased risk of HNC (Bottalico et al., 2011; Pierce Campbell et al., 2013; Torres et al., 2015; Agalliu et al., 2016; Donà et al., 2016; Nunes et al., 2016). Prevalence and concordance studies at cutaneous and mucosal sites also suggest a sexual transmission route, and that fingernails may be a source of autoinoculation of β-HPVs from skin to the oral cavity (Hampras et al., 2017; Winer et al., 2019). In this context, particular mention should be made of HPV types belonging to the species β-3, especially HPV-49, -75, and -76. They display transforming properties in primary human keratinocytes comparable to those of HPV-16 (Cornet et al., 2012; Minoni et al., 2020). Most importantly, some β-3-HPV types retain the ability to induce p53 degradation with a similar mechanism to HPV-16 (Cornet et al., 2012; Minoni et al., 2020). Moreover, HPV-49 E6 and E7 transgenic mice models show higher susceptibility to upper digestive tract cancers, with a molecular signature mimicking the damage induced by tobacco exposure (Viarisio et al., 2016, 2019).

## Discussion

In light of these recent lines of evidence, we believe that the conventional criteria for tropism classification need to be reconsidered. The assignment of PV types to genus and species of the phylogenetic tree is based merely on the nucleotide sequence of L1 gene and should not be assumed to reflect biological features nor to provide any definitive tropism information (de Villiers, 2013; Minoni et al., 2020). Recent epidemiological and experimental data on β-HPVs are in line with this hypothesis, and this is evident also for genus α, which groups both cutaneous and mucosal HPVs (Minoni et al., 2020). Therefore, the characterization of biological properties, beyond the comparative analysis of genetic sequences, appears to be necessary for a comprehensive evaluation of viral tropism, as shown also by studies of animal PVs (Altamura et al., 2016, 2018a; Alloway et al., 2020; Minoni et al., 2020). Moreover, the search for each human and animal PV should be expanded across a wide spectrum of anatomical sites, regardless of which body region they were initially isolated from, because limiting to one site may lead to an underestimation of their oncogenic potential at additional locations, even those coated by different types of epithelium.

Some evolutionary considerations should encourage colleagues to welcome our dissertation. It is well-known that completion of PVs life cycle with production of viral particles is strictly dependent on terminal differentiation of keratinocytes, thus productive infections of mucosal epithelia, which undergo incomplete keratinization, release few amounts of mature virions compared to those of the skin (Cubie, 2013). However, this in turn provides an advantage in terms of evasion of immune surveillance and viral persistence, therefore it would plausible that some cutaneous PVs evolved to exert also mucosal tropism in an attempt to establish a trade-off between efficient virus production and longer persistent infection. The recent development of MmuPV-1 infection models offers a significant opportunity to unravel the molecular mechanisms underlying these peculiar tropism features and other aspects of PVs biology impossible to study in classical transgenic mice (Spurgeon and Lambert, 2020).

We are confident that these considerations will be taken into account by scientists dealing with the issue of PVs tropism in the near future.

In conclusion, it is reasonable to foreshadow that the paradigm is going to fall. Therefore, researchers in the field should no longer just establish whether a PV is cutaneous *or* mucosal; rather, they should consider the possibility of it being cutaneous *and* mucosal.

## Author Contributions

GA, MT, and GB conceived the study and drafted the manuscript. All authors contributed to the article and approved the submitted version.

## Conflict of Interest

The authors declare that the research was conducted in the absence of any commercial or financial relationships that could be construed as a potential conflict of interest.
